# Quantitative behavioral evaluation of a non-human primate stroke model using a new monitoring system

**DOI:** 10.3389/fnins.2022.964928

**Published:** 2022-09-01

**Authors:** Toshikazu Hirohata, Takaya Kitano, Chizu Saeki, Kousuke Baba, Fumiaki Yoshida, Takashi Kurihara, Katsuhiro Harada, Shigeyoshi Saito, Hideki Mochizuki, Megumi Shimodozono

**Affiliations:** ^1^Department of Rehabilitation and Physical Medicine, Kagoshima University Graduate School of Medical and Dental Sciences, Kagoshima, Japan; ^2^Department of Neurology, Osaka University Graduate School of Medicine, Osaka, Japan; ^3^Academic Research Division, Department of Neurology, Faculty of Medicine, University of Toyama, Toyama, Japan; ^4^Department of Anatomy and Physiology, Faculty of Medicine, Saga University, Saga, Japan; ^5^Department of Pharmacology, Graduate School of Medical and Dental Sciences, Kagoshima University, Kagoshima, Japan; ^6^Division of Health Sciences, Department of Medical Physics and Engineering, Osaka University Graduate School of Medicine, Suita, Japan

**Keywords:** stroke, photothrombosis, non-human primate, behavioral analysis, Kinect

## Abstract

**Background:**

Recently, the common marmoset (*Callithrix jacchus*) has attracted significant interest as a non-human primate stroke model. Functional impairment in non-human primate stroke models should be evaluated quantitatively and successively after stroke, but conventional observational assessments of behavior cannot fully fit this purpose. In this paper, we report a behavioral analysis using MarmoDetector, a three-dimensional motion analysis, in an ischemic stroke model using photosensitive dye, along with an observational behavioral assessment and imaging examination.

**Methods:**

Ischemic stroke was induced in the left hemisphere of three marmosets. Cerebral infarction was induced by intravenous injection of rose bengal and irradiation with green light. The following day, the success of the procedure was confirmed by magnetic resonance imaging (MRI). The distance traveled, speed, activity time, and jumps/climbs were observed for 28 days after stroke using MarmoDetector. We also assessed the marmosets’ specific movements and postural abnormalities using conventional neurological scores.

**Results:**

Magnetic resonance imaging diffusion-weighted and T2-weighted images showed hyperintense signals, indicating cerebral infarction in all three marmosets. MarmoDetector data showed that the both indices immediately after stroke onset and gradually improved over weeks. Neurological scores were the worst immediately after stroke and did not recover to pre-infarction levels during the observation period (28 days). A significant correlation was observed between MarmoDetector data and conventional neurological scores.

**Conclusion:**

In this study, we showed that MarmoDetector can quantitatively evaluate behavioral changes in the acute to subacute phases stroke models. This technique can be practical for research on the pathophysiology of ischemic stroke and for the development of new therapeutic methods.

## Introduction

Stroke is a frequent and serious disease worldwide (GBD 2019 Stroke Collaborators, 2021). The majority of cerebrovascular accident patients who survive the acute phase of the disease are unable to perform activities of daily living due to residual disabilities such as motor paralysis, thus placing a heavy burden on the social economy. Animal experiments have greatly contributed to the pathophysiology of ischemic stroke and the development of new therapeutic methods. Most preclinical investigations designed to develop stroke therapies rely on the use of rodents (mice and rats) (Fisher et al., 2009). However, rodent models rapidly recover from motor paralysis after a stroke, whereas human stroke recovery generally takes several months after onset (Carmichael, 2005; Fan et al., 2017). This difference comes from differences in the brain structures and extent of brain damage. Therefore, it is more appropriate to use non-human primates as disease models in which the neuroanatomical structure is similar to that of humans (Fisher et al., 2009). Recently, there has been considerable interest in the common marmoset (*Callithrix jacchus*) in neuroscience research (Tokuno et al., 2012). Marmosets are small non-human primates; adults reach an average height of 20–30 cm and an average weight of 350–400 g, making them advantageous for laboratory use (Schiel and Souto, 2017). Furthermore, the establishment of genetic technologies, including marmoset transgenesis, has increased the value of marmosets as animal models for human diseases (Sasaki et al., 2009).

We previously established a stroke model in common marmosets using a photosensitive dye and observed the restoration process for 28 days (Ikeda et al., 2013). With the observational behavioral assessment employed in this study, the function of the forelimb and hindlimb of the paralyzed side decreased immediately after the infarction. The hindlimb recovered 3–4 days after the operation, while the forelimb symptoms persisted for 28 days after the procedure. Other studies have also reported that forelimb dysfunction persists a few weeks after onset (Freret et al., 2008; Bihel et al., 2011). However, some limitations and difficulties exist in assessing functional impairment in primate models of cerebral infarction using only conventional observational behavioral assessments. First, marmosets are very sensitive to stress and need to be handled in a stress-free manner during certain behavioral tests (Prins et al., 2017). Second, it is essential to establish a trusting relationship between evaluators and animals, and sufficient training must be provided beforehand (Le Gal et al., 2018). These two factors are major barriers to assessing functional impairment in primates. Therefore, there is a need for a simple, objective, and reproducible method to evaluate neurological deficits in stroke models. A new monitoring system, MarmoDetector, can automatically generate three-dimensional (3D) trajectories of marmoset behavior under stress-free conditions and analyze their movements over time (Yabumoto et al., 2019). We hypothesized that this system could be used to quantitatively assess acute behavioral changes in a common marmoset stroke model. In this paper, we report a behavioral analysis using MarmoDetector in an ischemic stroke model using a photosensitive dye, along with imaging examinations and observational behavioral assessment.

## Materials and methods

### Animals and housing

A total of three mature (original ID, age: H122, 4 years old; H123, 4 years old; 0506, 16 years old, respectively) wild-type female marmosets (280–390 g) were used. All marmosets were caged in an environmentally controlled room with a 12/12 h light/dark cycle and received disinfection treatment once daily. The temperature was set at 25.5–27.5°C. Items, such as bars and tubes, were placed in cages to enhance the environment of the animals. The animals were fed primate food *ad libitum* under the supervision of veterinarians, and a diet consisting of plums, mealworm larvae, grains, and eggs was provided twice a day. Water was provided *ad libitum* to all the animals. All animal experiments were conducted in accordance with the National Institutes of Health Guide for the Care and Use of Laboratory Animals (NIH Publication No. 8023, revised 1978). Every effort was made to minimize the number of animals used and their suffering. All animal experiments were approved by the Institutional Animal Care and Use Committee of Osaka University Graduate School of Medicine (approval number:01-056-003) and the Animal Experimentation Committee of Kagoshima University (approval number: MD19099, MD20026, and MD20087).

### Induction of cerebral infarction

**FIGURE 1:**
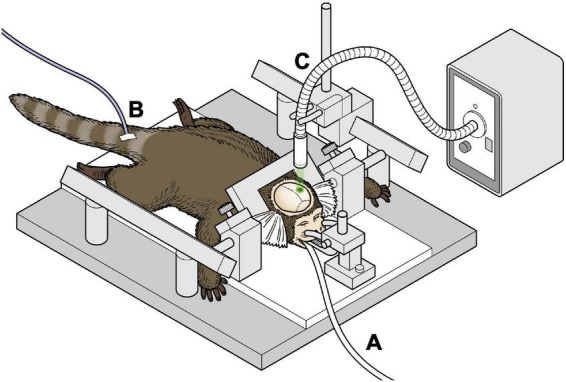
Schema of the surgical procedure of inducing infarction **(A)** marmosets were anesthetized with isoflurane and ventilator-controlled. **(B)** An intravenous catheter was placed in the tail vein and fluids were administered continuously. **(C)** The marmoset underwent stereotactic craniotomy, and green laser was irradiated on the exposed brain.

All marmosets were induced to have left hemisphere ischemic stroke. Details of the procedure for the induction of ischemia have been described in our previous study (Ikeda et al., 2013). An overview of the experiment is shown in Figure 1. Briefly, after intramuscular injection of atropine (0.05 mg/kg) and carprofen (3.75 mg/kg) as premedication, anesthesia with isoflurane (3–4% at induction, 0.5–2.5% maintenance) was performed. The airway was secured using an original intubation tube and the animal was managed with a ventilator. A warming mat with temperature compensation was used intraoperatively. Cefovecin (Convenia, Zoetis JP) was used as the intraoperative and postoperative antibiotic. A midline skin incision was made on the head, and the skull was carefully drilled to make 2.3 mm diameter hole above the target brain area to avoid damaging the dura mater. The sensory-motor cortex was defined according to the NIH marmoset brain atlas (Woodward et al., 2018) and initially set at 6 mm lateral to the midpoint and 9 mm anterior to the ear bar, and was also confirmed by electrophysiological mapping. The optic fiber was then placed just above the sensory-motor cortex connected to the laser light source (COME2 system: Lucir, Toyonaka City, Japan, or Z1M18B-F-532-pz: Z-laser, Freiburg, Germany). A 10° tilt was made to fit the brain surface vertically. To induce cerebral infarction, rose bengal (20 mg/kg) was administered intravenously and green light (532 nm) with a diameter of 4 mm was irradiated for 20 min at a light intensity of 20 mW. All surgeries were performed under general anesthesia with isoflurane. The skin was then sutured using absorbable sutures.

### MarmoDetector

**FIGURE 2:**
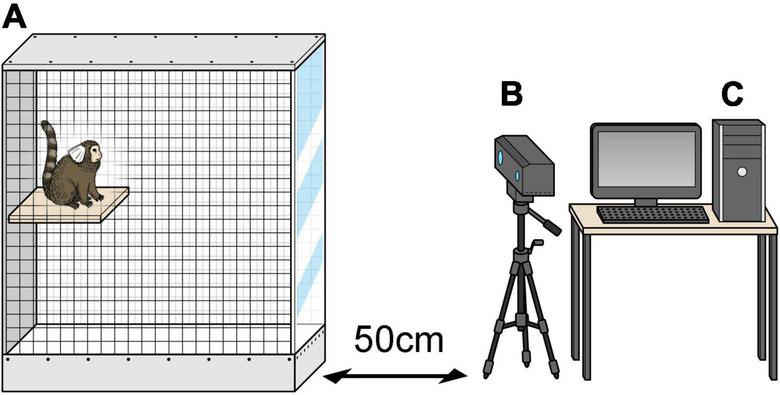
MarmoDetector **(A)** the cage was 525 mm long, 356 mm wide, and 776 mm high. **(B)** Video camera was placed 50 cm away in front of the experimental cage. **(C)** Personal computer including the software module. The three-dimensional location of the marmoset was recorded and analyzed.

MarmoDetector is a multimodal system that tracks the 3D coordinates of the marmoset, and the mechanism by which it accurately measures the position of a marmoset has been described in detail in previous studies (Yabumoto et al., 2019). A Kinect camera (Microsoft Kinect Sensor for Xbox One) was placed 50 cm from the experimental cage (Figure 2). The Kinect camera is equipped with an infrared (IR) camera and a depth sensor to visualize the entire cage and obtain a complete picture of the marmoset’s activities. The depth sensor of the Kinect device acquired depth data for the animals at five frames per second. The cage was 525 mm long, 356 mm wide, and 776 mm high and was designed to be large enough for marmosets to move around freely.

We designed a before-and-after comparison study in which each animal was compared in a healthy state and a post-stroke state. The healthy state was defined as the state in which the animals were not subjected to any invasive procedure, and the marmosets were allowed to adapt to the experimental cage for at least 16 h and recorded from 10:00 to 16:00 for 3 days. Postoperative evaluation began on the second day after stroke and was recording was conducted 10:00 to 16:00 until 28 days after surgery. The 3D location of the marmoset was recorded at five frames per second (i.e., 200 ms intervals). Then, the distance the marmoset moved in a day, speeds for each moment (only while the marmoset was moving), and activity time were analyzed. The postoperative measurements were calculated as a ratio using the healthy condition as baseline. In addition, we detected frames in which the marmosets were advancing vertically above 120 cm/s and calculated the number of such frames for each day. Additionally, we examined the trajectories of behavioral patterns exhibited by post-stroke marmosets in the cage.

### Observational behavioral assessment

We conducted conventional neurological examinations in addition to using MarmoDetector. The scale used in the experiment by Marshall and Ridley (1996) was adopted as a conventional method for behavioral assessment. This evaluates the presence or absence of abnormalities in a particular movement or posture through observation over a series of days. The evaluator observed the behavior of the marmosets in the cage for 3–5 min per day for the presence of four abnormal signs on each side of the body. These included: (1) hands and feet slipping off poles, (2) hands left dangling below the level of the perches, (3) an uncharacteristic posture in which the monkeys held their arms across the midline of their chest, and (4) an uncoordinated arm movement and hand waving. The monkeys were coaxed to move around the cage for marshmallow rewards to help them exhibit these specific effects. One point was added for each sign for a total score of 4 points. This evaluation was performed pre- and postoperatively for 28 days after stroke. In this study, we did not employ a working test such as the Hill and Valley Staircase test to put as little stress on the marmosets as possible.

### Magnetic resonance imaging scan

Magnetic resonance imaging (MRI) (7T; PharmaScan 70/16 US; Bruker Biospin, Ettlingen, Germany) of the brain with a 60 mm volume coil was performed on postoperative day 1. General anesthesia with isoflurane (3–4% at induction, 0.5–2.5% at maintenance) was used. The marmosets were positioned in a specially designed stereotaxic frame with mouth and ear bars to prevent movement during the acquisition. The body temperature was maintained at 36.5°C with regulated water flow and continuously monitored using a physiological monitoring system (SA Instruments, Inc., Stony Brook, United States). All processes, including preparation, were completed within 30 min. MRI examinations were performed using multi-shot echo planar diffusion-weighted image (DWI, 30 diffusion directions, *b*-values = 650 and *b* = 0 s/mm^2^, echo time [TE]/repetition time [TR] = 20/2000 ms, matrix = 96 × 96, field of view [FOV] = 51.2 × 51.2 mm, 30 slices, slice thickness = 1.0 mm, number of average = 2, segment = 4 segments, scan time = 9 min 20 s, and T_2_-weighted imaging (rapid acquisition with relaxation enhancement [RARE] factor = 8, TE/TR = 33/3200 ms, matrix = 256 × 256, FOV = 51.2 × 51.2 mm, 30 slices, thickness = 1.0 mm, number of average = 4). Infarct volumes were derived from diffusion-weighted images of high-intensity signal areas.

### Statistical analysis

Since the distance traveled and the median travel speed vary greatly among individuals, they are measured before and after stroke induction and shown as ratios (the *y*-axis of the graph is the postoperative/preoperative value). Differences between groups were examined using a paired *t*-test. Correlations between evaluations based on MarmoDetector and conventional neurological scores were analyzed using Spearman’s correlation analysis. Statistical significance was set at *P* < 0.05. All analyses were performed using GraphPad Prism (Prism9, San Diego, CA, United States).

## Results

### Infarcts

**FIGURE 3:**
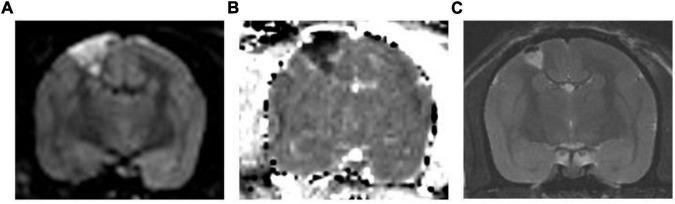
Representative MR Images. **(A)** Diffusion-weighted image and **(B)** corresponding apparent diffusion coefficient map. The high-intensity area in the diffusion-weighted image shows low intensity in the apparent diffusion coefficient map, which suggests that this area is infarcted. **(C)** T2-weighted image 1 month after surgery. The infarcted area shows hyperintensity in the T2-weighted image.

Using MRI, we confirmed successful induction of cerebral infarction. High-intensity areas were consistently observed on MRI diffusion-weighted images, and these areas showed low intensity on the apparent diffusion coefficient maps (Figure 3) in all three animals. These areas include the motor cortex, as described in the atlas (Palazzi and Bordier, 2008). The mean length diameter was 4.8 mm and the mean width diameter was 2.8 mm in the axial image. Coronal imaging revealed that the mean infarct was 3.1 mm in depth. The mean infarct volume was found to be 28 mm^3^. The infarcted areas showed hyperintensity on T_2_-weighted images 1 month after surgery.

### Evaluation based on MarmoDetector

**FIGURE 4:**
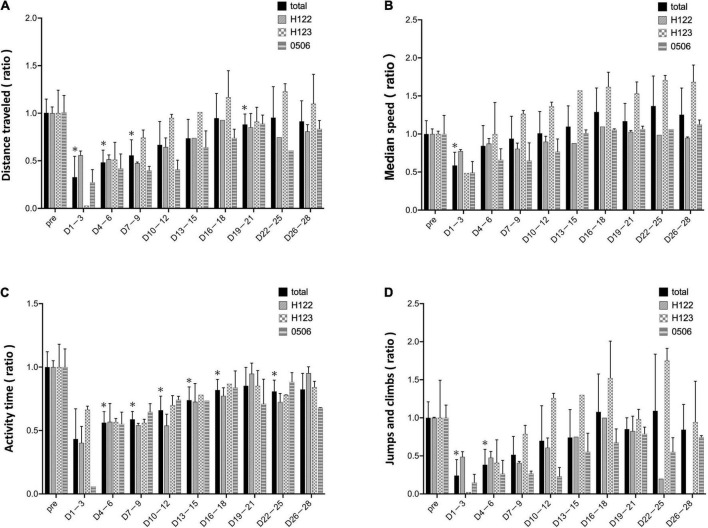
Evaluation based on MarmoDetector The bar shows data from each animal in individual lines besides total. **(A)** *Y*-axis indicates the ratio of preoperative/postoperative distance traveled. The distance traveled dropped immediately after the stroke, and gradually improved, recovering to the same level as that before the stroke at 16–18 days after stroke. **(B)** *Y*-axis: the ratio of preoperative/postoperative median movement speed. The travel speed dropped immediately after the stroke, and then significantly improved on days 4–6, recovering to the same level as that before the stroke on days 10–12. **(C)** *Y*-axis: the ratio of preoperative/postoperative activity time. Activity time had improved slightly after stroke. **(D)** *Y*-axis: the ratio of preoperative/postoperative jumps/climbs. Jumps/climbs is the number of frames that moved vertically above 120 cm/s. Jumps/climbs tend to decrease during the first postoperative week, but the recovery process appears to vary from individual to individual. Postoperative values are shown as means and standard errors for every 3 days. *P < 0.05 in comparison with preoperative values indicating the presence of deficits (paired *t*-test).

**FIGURE 5:**
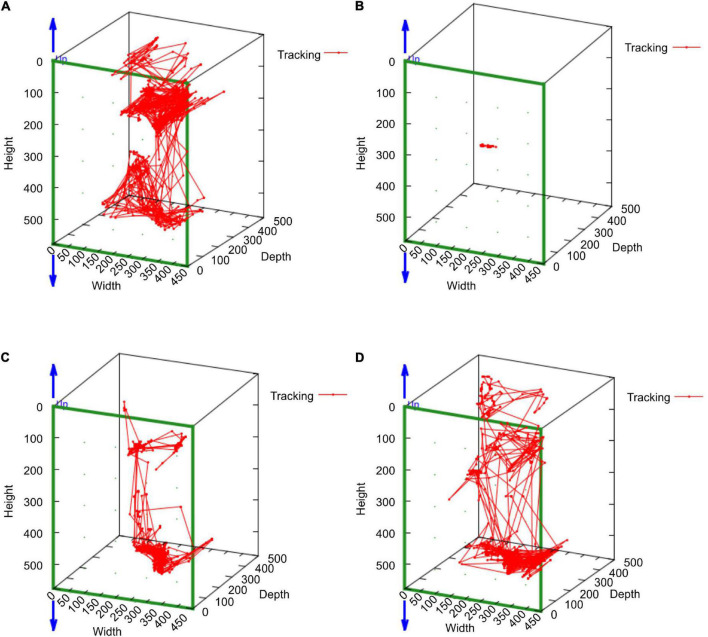
The MarmoDetector analyses the trajectory of the marmoset in action Behavioral trajectory at a certain time: The marmoset (H123) was active in the cage at **(A)** the pre-operation stage. The range of moving trajectory was considerably limited on **(B)** the second postoperative day. It started enlarging on **(C)** the third postoperative day and **(D)** the fourth postoperative day.

In the mean of the data for the three marmosets, distance traveled and median speed significantly dropped after surgery (Figures 4A,B). The distance traveled gradually improved in all marmosets and recovered to the same level as that before the stroke on days 16–18 after stroke (Figure 4A). Median travel speed dropped immediately after the stroke and then improved significantly on days 4–6, recovering to the same level as before the stroke on days 10–12 (Figure 4B). As shown in the distance traveled, activity time dropped after surgery and recovered gradually and reached to the preoperative level on days 19–21 (Figure 4C). Jumps/climbs tended to decrease during the first postoperative week, but the recovery process appeared to vary from individual to individual (Figure 4D). The range of moving trajectory was considerably limited on the second postoperative day, but it started enlarging on the third postoperative day (Figure 5).

### Observational behavioral assessment

**FIGURE 6:**
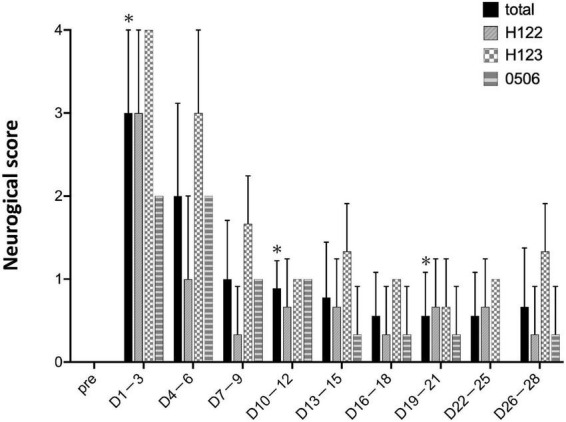
Neurological scores. Postoperative values are shown as means and standard errors in every 3 days. *P < 0.05 in comparison with preoperative values indicating the presence of deficits (paired *t*-test).

As a matter of course, the preoperative neurological score was zero for all individuals (Figure 6). Within 1–3 days after stroke, the neurological score was the highest and the marmosets barely ate at all. The scores were high until 4–6 days post stroke. After day 7, the marmosets recovered and fed themselves by the upper limb on the non-paralyzed side. Neurological scores did not recover to the pre-infarction level during the observation period (28 days).

### Correlation between evaluation by MarmoDetector and conventional neurological scores

**FIGURE 7:**
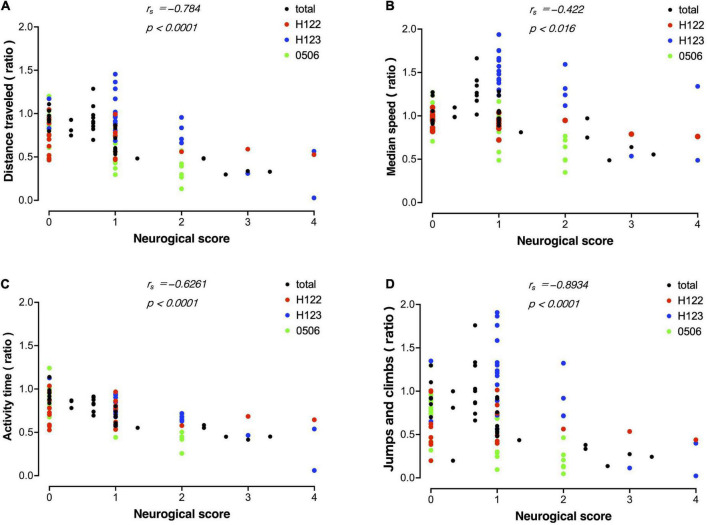
Correlation between evaluation by MarmoDetector and conventional neurological scores Scatter plot discriminates among data from individual animals besides total. **(A)** Scatter plot of distance traveled and neurological score. *X*-axis: neurological score, *Y*-axis: preoperative/postoperative distance traveled. **(B)** Scatter plot of median speed and neurological score. *X*-axis: neurological score, *Y*- axis: preoperative/postoperative median speed. **(C)** Scatter plot of activity time and neurological score. *X*-axis: neurological score, *Y*- axis: preoperative/postoperative activity time. **(D)** Scatter plot of jumps/climbs and neurological score. *X*-axis: neurological score, *Y*- axis: preoperative/postoperative activity time. Both indices were significantly correlated with the neurological score. Correlation analysis was performed using Spearman’s rank correlation coefficient to obtain the correlation coefficient r_s_ and *P*-value.

A correlation analysis was performed between measurements using MarmoDetector and the conventional assessment of neurological scores. The measurements and scores included in the correlation analysis were the mean of data sets from the three animals. There were significant correlations between the distance traveled and neurological score (Figure 7A), between the median movement speed and neurological score (Figure 7B), between activity time and neurological score (Figure 7C), and between Jumps/climbs and neurological score (Figure 7D).

## Discussion

Many effective stroke treatment strategies in animal studies result in failed clinical trials (Dirnagl and Fisher, 2012).

An important contributing factor is the inadequacy of the stroke model. Rodents are the most common animals used in stroke research, but genetic differences and species have significant impacts on stroke treatment (Wu et al., 2021). The Stroke Therapy Academic Industry Roundtable [STAIR] (1999) recommends that preclinical stroke research should be conducted in multiple animal species, including non-human primates, and in animals of various ages and comorbidities. Among them, marmosets have several advantages over rodents and large primates, and are a promising strategy for the study of stroke pathophysiology and treatment.

This study first focused on creating a protocol for a photothrombotic stroke model to reduce invasive procedures on animals as much as possible. In our preliminary experiments, marmosets were induced to have left hemisphere ischemic stroke with a light intensity of 300 mW, similar to that employed in our previous experiment (Ikeda et al., 2013). They could occasionally cause unlocalized infarction and postoperative brain edema. This unwanted enlarged infarction increased the risk of severe symptoms, such as severe hemiparesis, causing frequent falls in the cage. This symptom requires intensive care for the marmoset, which could affect any behavioral experiment and create a bias in the effectiveness of treatment for stroke. In the present study, we made two major improvements to the procedure for creating cerebral infarction from our previous study (Ikeda et al., 2013), in order to reduce invasiveness and increase reproducibility. First, we made a burr-hole of 2.3 mm diameter in the skull to reduce scatter of the green light which could cause collateral brain damage. Second, the tilt angle was strictly set vertical to the brain surface to avoid infarction on the contralateral side. Furthermore, one marmoset was sacrificed for the purpose of testing various light parameters, and histological confirmation of the infarct area. Thus, we established a method to localize cerebral infarction to preserve the function of the non-target side. This allowed us to observe the animals from a very early stage after the onset of infarction. The sample size of three marmosets might be relatively small compared with other general stroke studies in rodents. But the number of animals is not statistically problematic, which is the result of efforts to minimize the sample size of precious non-human primates through appropriate protocols.

Our improved photothrombosis method has better consistency as a stroke model than conventional methods. This was confirmed by MRI-based imaging evaluation and phenotypic assessment using MarmoDetector. In recent years, the review (Uzdensky, 2018) and excellent practical guidance (Labat-gest and Tomasi, 2013; Talley Watts et al., 2015; Song et al., 2016) have been published on photothrombotic stroke models. Photothrombotic stroke causes endothelial damage and platelet aggregation in vessels, resulting in distinct ischemic lesions in the cerebral cortex (Watson et al., 1985). Because of this intra-arterial occlusion, ischemic cell death progresses rapidly (Dietrich et al., 1986). Therefore, penumbra and collateral channel formation and reperfusion rarely occur (Lee et al., 1996). Research on changes in sensory-motor cortex mapping in response to stroke has exploited this feature (Harrison et al., 2013). This research used a photothrombotic stroke model to examine sensorimotor cortex mapping before and after stroke, noting that changes in cortical map structure occur after stroke, as survival regions take over function. While this is likely to cause difficulties in other stroke models, which are more varied owing to the progression of collateral tracts and the formation of the penumbra, the photothrombotic model is well suited to the study of functional reorganization in the cortex. The photothrombotic stroke model can also accurately define the ischemic region based on the size of the exposed area. This was applied to induce cerebral infarction in the internal capsule and caudoputamen by stereotaxic fixation of an optical fiber (Kuroiwa et al., 2009; Kim et al., 2014). Additionally, photothrombosis is less invasive and has a higher survival rate. The eldest marmoset used in this experiment was 16 years old. The parameter of age may have affected the stroke recovery process, but the small sample size made it difficult to correlate age with the extent of stroke. But even the eldest marmoset did not show any complications during the experiment. If marmosets survive long after stroke onset and the area of the stroke is confirmed by MRI, the photothrombotic method can be used as a chronic stroke model. Elderly animals are presumably ideal for stroke research because most stroke patients are also aged. Thus, the low invasiveness of the proposed method is an important feature.

In this study, we evaluated the acute phase of a non-human primate stroke model using the 3D analysis software, MarmoDetector. We previously analyzed the motion of a similar stroke model by using another motion analysis system (DIPP-Motion Pro) (Ikeda et al., 2013). However, this analysis is based only on scenes of feeding behavior, which limits the analysis time and range of behavior. In the current study, automatic recording covers 6 h from 10:00 to 16:00 for 28 days after stroke. Since animals fluctuate greatly in their behavior within and between days, long-term recording of the amount of movement for most of the daytime is a very useful means for minimizing data variability. In addition, the measurement is performed automatically by the system, so human measurement bias can be minimized.

We have previously reported that MarmoDetector provides the precise 3D location of marmosets (Yabumoto et al., 2019). In the current study, the amount and median speed of movement measured by MarmoDetector showed a significant decrease immediately after stroke. In the acute phase of stroke, marmosets exhibit clear signs of weakness and poor feeding activities. Even if the marmoset rejects behavioral tests because of these stresses, the speed and distance of movement can be measured simply and is useful for quantitatively assessing the acute state of cerebral infarction. Furthermore, unlike other behavioral tests, marmosets do not need to be trained in advance. This overcomes several barriers in behavioral testing. However, behavioral testing has several limitations. First, there are individual differences in adapting to the training. Second, behavioral tasks after stroke have a learning effect that leads to functional recovery (Le Gal et al., 2018). These two factors can be major problems when performing comparative control studies.

In non-human primate stroke models, neurological scores are useful as a test of sensorimotor function (Marshall and Ridley, 1996). In 1996, Marshall and Ridley published a neurological score adapted to marmosets, after which this neurological score has improved and several variations have been developed (Virley et al., 2004; Freret et al., 2008; Bihel et al., 2010, 2011; Ikeda et al., 2013; Puentes et al., 2015). These methods are among the most frequently used in non-human primate stroke models. Marmosets subjected to intraluminal permanent occlusion of the middle cerebral artery have significantly detected functional deficits based on neurological scores up to 41 days post-stroke at the end of the observation period (Bihel et al., 2010, 2011). Other models show significant signs in neurological score up to 10 days in the permanent occlusion of the anterior choroidal artery model (Puentes et al., 2015) and up to 28 days in the photothrombosis model (Ikeda et al., 2013). A recently reported model of cerebral infarction induced by stereotactic injection of the mitochondrial toxin malonate showed long-term motor deficits 12 weeks after surgery (Le Friec et al., 2021). In the present study, the amount of movement, along with the neurological score, showed a trend toward recovery from immediately to 14 days after stroke onset. The analysis showed a similar tendency between the amount of movement and neurological score, indicating that the amount of movement can be used as an alternative method to the neurological score.

There is no precedent for MRI evaluation of photothrombotic stroke models in marmosets, although studies on MRI evaluation have been reported in rhesus monkeys (Zhang et al., 2021). The MRI results showed decreased edema volume on postoperative day 14. Infarcts with long T2 and high diffusion-weighted signal development were observed in the target area on postoperative days 1–63. MRI in the current study showed that brain edema disappeared 1 month after surgery, similar to previous studies (Zhang et al., 2021). We had difficulty correlating infarct regions with motor impairment due to the small sample size. Histopathological analysis revealed persistent damage to the cerebral cortex in a photothrombotic stroke model (Zhang et al., 2021). It would be difficult to correlate infarct volume with motor impairment because small thrombi in the distal vessels could not be detected on MRI. We did not evaluate each animal with MRI before cerebral ischemia. This is because administering general anesthesia many times in a short period affects the animal’s health and behavior.

We examined the trajectories with the MarmoDetector to assess behavioral patterns after stroke. We found that the preoperative and the postoperative behavioral patterns in the cage had changed significantly. In addition, as shown in the graph, the number of jumps decreased considerably after surgery. Marmosets have a habit of climbing to high places, but the disability caused by stroke reduced their ability to jump and climb cage walls. We used MarmoDetector to analyze the behavior of the stroke model; however, this method can also be applied to other disease models. In studies on marmoset sleep (Hoffmann et al., 2012) and Parkinson’s disease (Choudhury and Daadi, 2018), a small device was placed on the marmoset’s head to analyze locomotion. This situation is unfamiliar to marmosets and may affect their behavior. MarmoDetector uses IR monitoring to capture movement accurately, even in dark environments. MarmoDetector can detect behavioral changes in psychiatric and neurological disorders as well as the therapeutic effects and side effects of new drugs. However, the Kinect sensor used in MarmoDetector focuses on capturing the position of the marmoset, which may limit its ability to record fine and involuntary movements of the distal limb. Further development is required to broaden such applications.

The evaluation methods used in this study have made it possible to extract highly reproducible data in a cerebral infarction model with a uniformly stable infarction site, which has been difficult to perform in non-human primates. Adding histological and electrophysiological evaluations to this method will help elucidate the neural mechanisms and neuroplasticity that contribute to stroke recovery and to validate new treatments for stroke.

## Conclusion

This study showed that MarmoDetector can quantitatively evaluate behavioral changes in the acute to subacute phases of stroke models. This technique is practical for research on the pathophysiology of ischemic stroke and for the development of new therapeutic methods.

## Data availability statement

The original contributions presented in this study are included in the article/Supplementary material, further inquiries can be directed to the corresponding authors.

## Ethics statement

The animal study was reviewed and approved by the Institutional Animal Care and Use Committee of Osaka University Graduate School of Medicine (approval number: 01-056-003) and the Animal Experimentation Committee of Kagoshima University (approval numbers: MD19099, MD20026, and MD20087).

## Author contributions

KB, FY, TKu, and KH designed the experiment. TH, TKi, CS, KB, FY, and TKu performed the stroke surgery, behavioral experiments, and data analysis. TH and TKi wrote the manuscript. SS performed the MRI scan. HM and MS supervised all experiments and analyses. TH, TKi, CS, KB, FY, TKu, HM, and MS discussed the experiments and edited the manuscript. All authors contributed to the article and approved the submitted version.
