# Supplementary material for: Quantitative behavioral evaluation of a non-human primate stroke model using a new monitoring system
Source: Front Neurosci. 2022 Sep 1;16:964928. doi: 10.3389/fnins.2022.964928 (PMC9475201; doi:10.3389/fnins.2022.964928)

Appendix A. Supplementary data

Supplementary figure Legends.

Supplementary Figure S1.

MR images in each marmoset. The infarcted area shows hyperintensity in the T2-weighted image. All marmoset have cerebral infarct images confined to the cortex.

Supplementary Figure S2.

MR image in a marmoset subjected to only isoflurane anesthesia. Isoflurane does not affect T2-weighted images.

Supplementary Figure S1.
MR images in each marmoset.

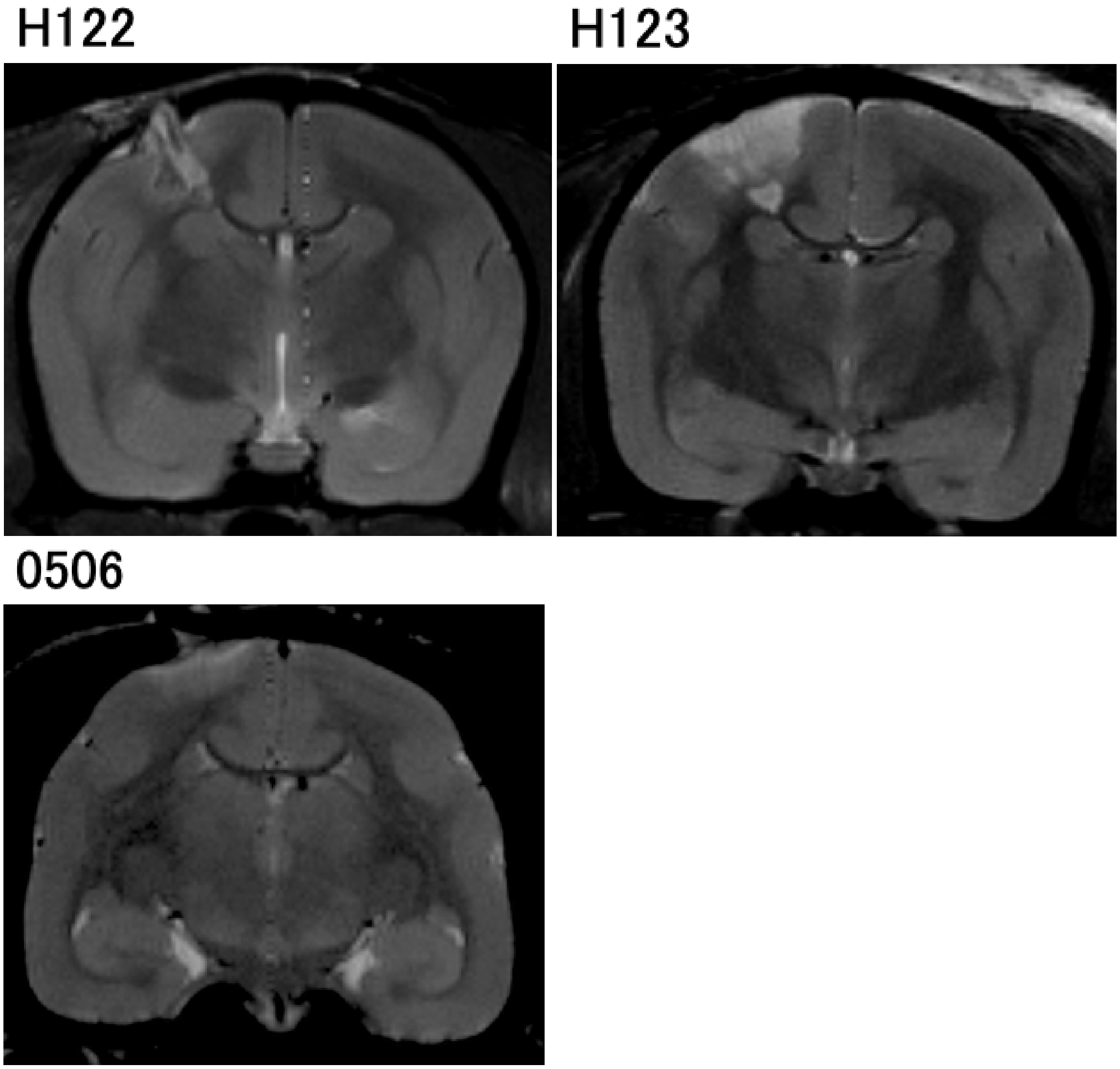

Supplementary Figure S2.
MR image in a marmoset subjected to only isoflurane anesthesia.

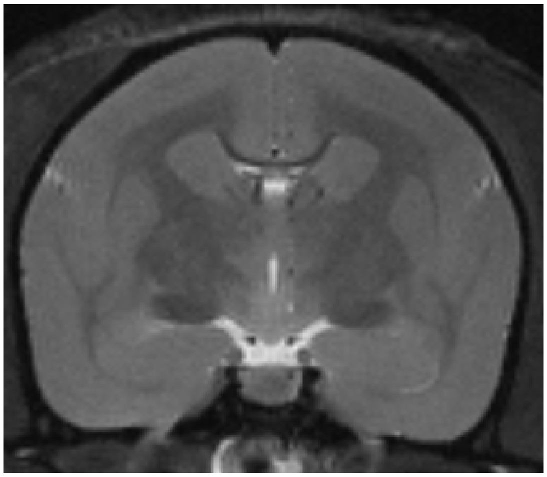

Supplement: Supplementary file 1 [file Data_Sheet_1.pdf]
